# Nonpharmacological Approaches to Managing Heart Failure With Preserved Ejection Fraction

**DOI:** 10.1161/CIRCHEARTFAILURE.123.011269

**Published:** 2024-06-18

**Authors:** Feiyang Tang, Haofu Han, Sheng Fu, Qiming Liu, Shenghua Zhou, Jiapeng Huang, Yichao Xiao

**Affiliations:** 1 Department of Cardiovascular Medicine, Second Xiangya Hospital (F.T., H.H., Q.L., S.Z., Y.X.), Central South University, Changsha, China.; 2 Xiangya School of Medicine (F.T., H.H.), Central South University, Changsha, China.; 3 Division of Cardiovascular Medicine, Department of Medicine (S.F.), University of Louisville, KY.; 4 Department of Anesthesiology and Perioperative Medicine (J.H.), University of Louisville, KY.

**Keywords:** denervation, heart failure, hypertension, vagus nerve stimulation

## Abstract

Heart failure with preserved ejection fraction (HFpEF) is a common subtype of heart failure marked by impaired left ventricular diastolic function and decreased myocardial compliance. Given the limited availability of evidence-based pharmacological treatments for HFpEF, there is a growing interest in nonpharmacological interventions as viable therapeutic alternatives. This review aims to explore the pathophysiology of HFpEF and present recent advancements in nonpharmacological management approaches, encompassing noninvasive therapies, invasive procedures and targeted treatments for comorbidities. An extensive literature review was undertaken to identify and synthesize emerging nonpharmacological treatment options for HFpEF, assessing their potential to enhance patient outcomes. Nonpharmacological strategies, such as vagus nerve stimulation, percutaneous pulmonary artery denervation, renal denervation, transcatheter insertion of atrial shunts and pericardial resection, demonstrate promising potential for alleviating HFpEF symptoms and improving patient prognosis. Moreover, addressing comorbidities, such as hypertension and diabetes, may offer additional therapeutic benefits. These cutting-edge techniques, in conjunction with well-established exercise therapies, pave the way for future research and clinical applications in the field. Nonpharmacological interventions hold promise for advancing HFpEF patient care and fostering a deeper understanding of these treatment approaches, which will facilitate new clinical applications and contribute to the development of more targeted therapies.

Heart failure (HF) is a condition in which the pumping function of the heart is abnormal under the influence of various clinical syndromes and cardiovascular diseases, resulting in a relative and absolute deficit in cardiac output with subsequent structural changes and functional decline in the body.^1^ The recently published universal definition of HF classified HF based on ejection fraction^2^: (1) heart failure with reduced ejection fraction (HFrEF): left ventricular ejection fraction (LVEF) ≤40%; (2) HF with mildly reduced ejection fraction: LVEF 41% ≈49%; (3) heart failure with preserved ejection fraction (HFpEF): LVEF ≥50%; and (4) HF with improved ejection fraction: baseline LVEF ≤40%, LVEF increase ≥LVEF>40% at the second measurement. Despite the availability of evidence-based pharmacological treatments, such as SGLT2 (sodium-glucose cotransporter 2) inhibitors and diuretics,^3^ the management of HFpEF remains challenging. This complexity can be attributed, in part, to the intricate pathophysiological mechanisms inherent to HFpEF and the prevalence of numerous coexisting comorbidities within this patient cohort.^4^ As a result, there is still a pressing need for the development of effective treatment strategies for HFpEF, which has led to increased interest in nonpharmacological interventions as potential therapeutic alternatives.

## PATHOPHYSIOLOGY OF HFpEF

HFpEF accounts for over half the number of HF patients. Although cardiac impairment of HFpEF mainly manifests as diastolic dysfunction due to myocardial hypertrophy, its pathophysiologic basis is often multifactorial, resulting in marked heterogeneity in its phenotypic manifestations.^5^ In contrast to HFrEF, HFpEF frequently affects older women and is associated with concomitant conditions such as anemia, metabolic syndrome, hypertension and diabetes.^6^ The underlying interactions contribute to the complexity of the pathogenesis of HFpEF and at least partially contribute to the lack of effective therapies for HFpEF.

**Figure 1.**
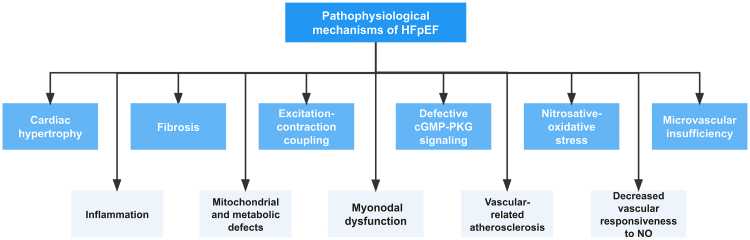
**Pathophysiological mechanisms underlying HFpEF.** This illustration demonstrates the intricate and multifaceted nature of HFpEF, which contributes to the limited efficacy of drug therapy alone, despite numerous clinical trials. However, ongoing research exploring nonpharmacological treatment approaches and their mechanisms may potentially mark a turning point in HFpEF management. This review examines nonpharmacological interventions and their associated mechanisms for HFpEF reported in the past year. cGMP indicates cyclic guanosine monophosphate; HFpEF, heart failure with preserved ejection fraction; and PKG, protein kinase G.

The pathophysiologic causes of HFpEF are numerous and complex (Figure 1).^7^ Left ventricular (LV) hypertrophy is a common phenotypic characteristic under which numerous cellular and molecular abnormalities occur, including improper calcium handling, cardiac fibrosis, oxidative stress, cell death, inadequate angiogenesis, mitochondrial dysfunction, metabolic reprogramming, cell growth and protein synthesis and the development of embryonic genetic programs.^8^ Likewise, myocardial interstitial fibrosis is also frequently found; meanwhile, some studies have shown that many fibrosis-related blood markers are associated with diastolic dysfunction in HFpEF,^9^ and interstitial fibrosis may bring about the formation of HFpEF by decreasing ventricular compliance and interacting with several other pathological processes. Similar to HFrEF, abnormal myocardial excitation-contraction coupling may also be involved in diastolic dysfunction in HFpEF^10^; however, experimental studies of live myocardial cell sampling from HFpEF patients are still lacking for further determination. On the contrary, frozen cardiomyocytes can be used to study abnormalities in the functional structure of myofascia itself. Some studies have indicated that reduced PKG (protein kinase G) activity may be associated with hypophosphorylation of related proteins on myofascia^11^ and ultimately lead to reduced ventricular compliance. Signaling pathways associated with PKG have also been linked to myocardial hypertrophy, fibrosis and angiogenesis and are potential therapeutic targets for HFpEF that are currently the focus of numerous studies. In addition, nitrosative-oxidative stress, microvascular insufficiency, epicardial lipids, inflammation and mitochondrial and metabolic defects have been indicated to be probably associated with HFpEF.

Extracardiac abnormalities such as vascular-related atherosclerosis and decreased vascular responsiveness to nitric oxide have also been shown to contribute to the pathophysiology of HFpEF.^12^ For example, aortic stiffness is now considered a prominent risk factor for cardiovascular disease^13^ and may play a role in HFpEF formation through its association with the progression of hypertension.

## NONPHARMACOLOGICAL MANAGEMENT APPROACHES

Nonpharmacological approaches encompass a diverse array of techniques, ranging from physical therapies and invasive procedures to targeted treatments for comorbidities. The exploration and development of these innovative strategies have the potential to significantly advance patient care for those with HFpEF and contribute to a better understanding of the condition’s complex nature. In the following sections, we will discuss various nonpharmacological management approaches, highlighting their potential benefits and applications in the treatment of HFpEF.

### Noninvasive Therapy

#### Vagus Nerve Stimulation

Vagal nerve stimulation (VNS) is a neuromodulation technique that regulates the functional activity of the vagus nerve via chronic microcurrent stimulation.^14^ Its prior clinical use has been mainly limited to the field of epilepsy. A recent study by Stavrakis et al^15^ found that chronic low-level transcutaneous vagus nerve stimulation significantly enhances cardiac function, reduces inflammation and improves quality of life in patients with HFpEF, showcasing potential therapeutic benefits without device-related side effects. The ANTHEM-HFpEF study (Autonomic Neural Regulation Therapy to Enhance Myocardial Function in Patients with Heart Failure and Preserved Ejection Fraction)^16^ demonstrates that autonomic regulation therapy, through cervical vagus nerve stimulation, is safe and improves symptoms, exercise tolerance, autonomic function and cardiac electrical stability in patients with heart failure with preserved or mildly reduced ejection fraction without changing mechanical cardiac functions. In HFpEF, activation of the vagus nerve may be able to attenuate the chronic proinflammatory state associated with the disease state as well as its numerous comorbidities.^17^ In studies by Pavlov^18^ and Wang et al^19^, VNS exerted significant anti-inflammatory effects in systemic inflammation as well as sepsis. Additionally, the application of VNS to patients with rheumatoid arthritis and atrial fibrillation requiring surgery has shown promising anti-inflammatory effects.^20,21^ Recently, low-level transcutaneous vagus nerve stimulation was shown to improve markers of diastolic function in a mature rat model.^22^ On this basis, the study by Elkholey^22,23^ investigated the effects and specific mechanisms of the anti-inflammatory effect of VNS. The results of the study showed that low-level transcutaneous vagus nerve stimulation resulted in increases in blood pressure, a reduction in left ventricular hypertrophy and improved LV strain while preventing the decline in diastolic function.

**Figure 2.**
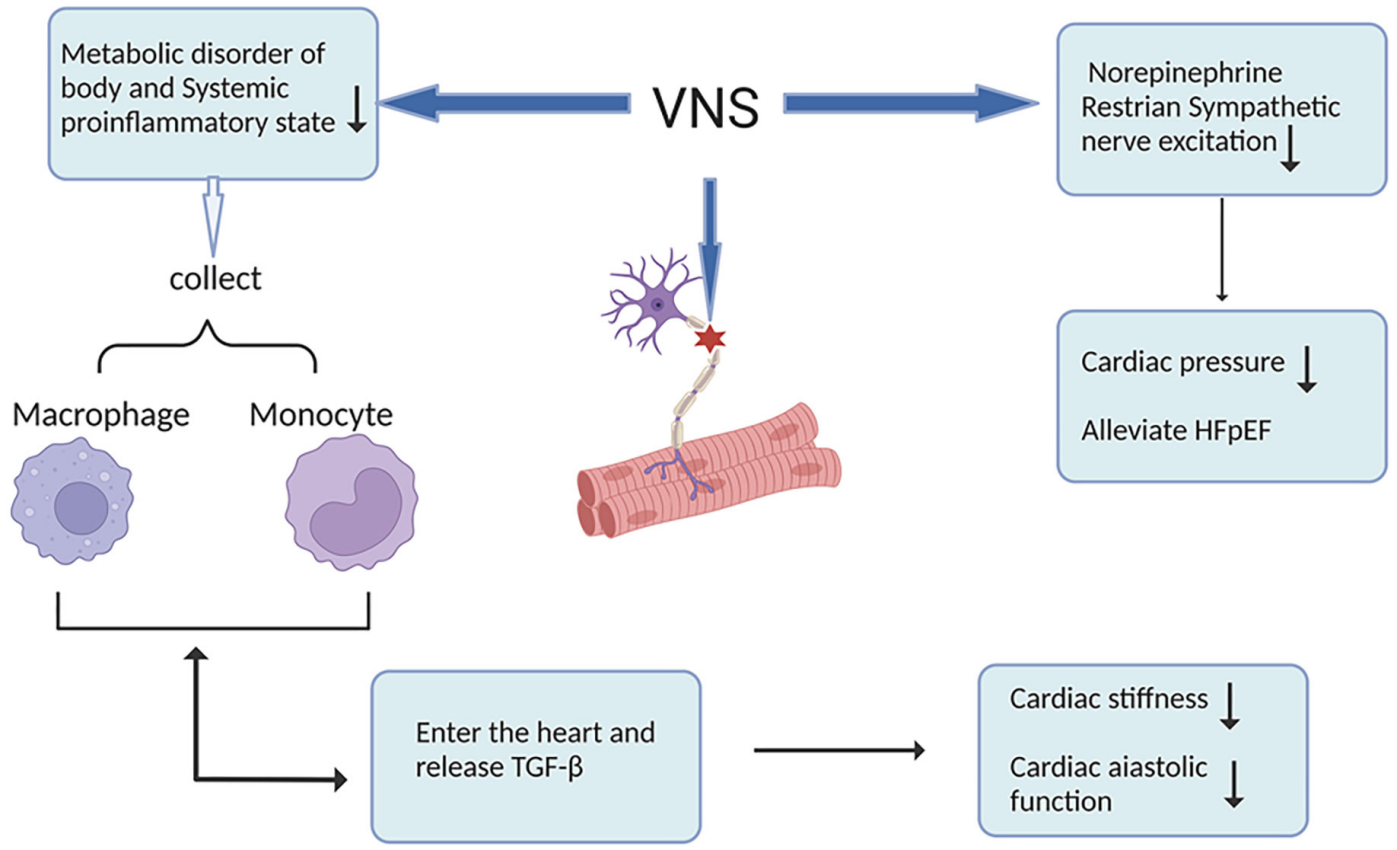
**Vagal nerve stimulation and norepinephrine for HFpEF management.** Vagal nerve stimulators reduce metabolic disorders and systemic proinflammatory states, while norepinephrine restores sympathetic nerve excitation, resulting in decreased cardiac pressure and stiffness, improved cardiac diastolic function, and HFpEF alleviation. HFpEF indicates heart failure with preserved ejection fraction; TGF-β, transforming growth factor-β; and VNS, vagal nerve stimulation. Created with Biorender.com.

Primary conditions such as hypertension and diabetes cause metabolic disturbances in the body, inducing a systemic proinflammatory state that leads to the recruitment of macrophages and monocytes and their entry into the heart. These inflammatory cells promote the differentiation of fibroblasts into myofibroblasts by releasing growth factors (TGF-β [transforming growth factor-β]) in the heart, especially in the left ventricle, thus promoting left ventricular fibrosis.^24^ This results in an increase in collagen content in the left ventricle and leads to an increase in myocardial fiber stiffness. Ultimately, this leads to diastolic dysfunction (in which increased sympathetic activity also plays an important role). VNS regulates inflammation in several ways. First, VNS inhibits sympathetic excitation.^25,26^ It attenuates the release of cardiac sympathetic norepinephrine, dilates cardiac microcirculatory vessels and increases microcirculatory tolerance in patients.^27,28^ This inhibitory effect attenuates the cardiac autonomic over-reflex response, resulting in a decrease in neurogenic proinflammatory factors. Meanwhile, ventricular pressure overload can cause increased expression of IL-18 (interleukin-18), IL-11, osteopontin gene and proteins, which were all found to be important factors in promoting cardiac fibrosis.^29–32^ VNS can inhibit the expression of myocardial proinflammatory genes, prevent the development of diastolic dysfunction and improve myocardial function^33^ (Figure 2).

#### Low-Intensity Pulsed Ultrasound

**Figure 3.**
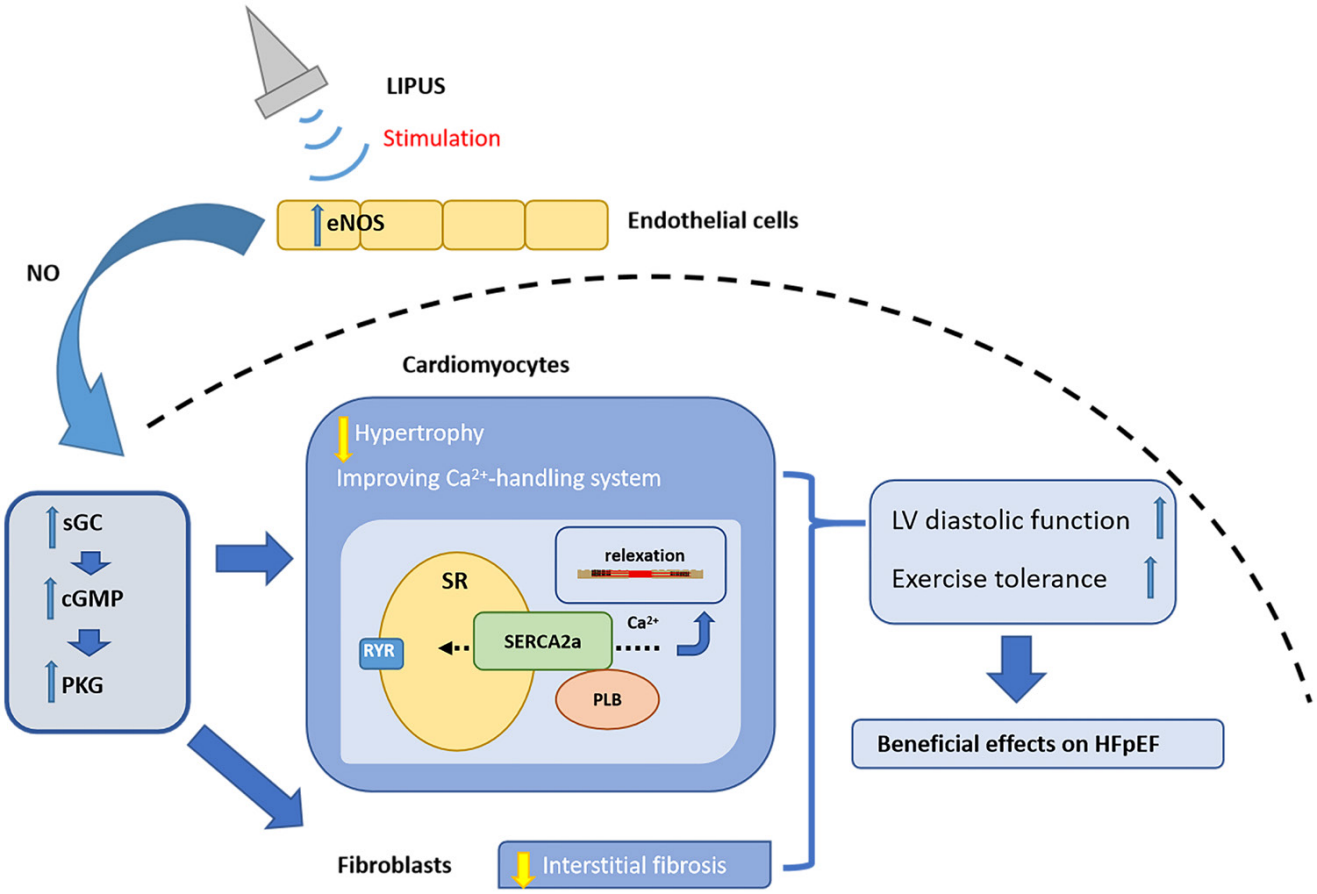
**LIPUS enhances myocardial calcium processing proteins and the treatment of HFpEF.** LIPUS improves myocardial calcium-related protein processing by stimulating the eNOS-NO-cGMP-PKG signaling pathway, which in turn modulates myocardial diastolic function and facilitates HFpEF treatment and recovery. cGMP indicates cyclic guanosine monophosphate; eNOS, endothelial nitric oxide synthase; HFpEF, heart failure with preserved ejection fraction; LIPUS, low-intensity pulsed ultrasound; NO, nitric oxide; PKG, protein kinase G; PLB, phospholamban; RYR, ryanodine receptor; SERCA2a, sarcoplasmic/endoplasmic reticulum Ca^2+^ ATPase 2a; sGC, soluble guanylate cyclase; and SR, sarcoplasmic reticulum. Created with Biorender.com.

Low-intensity pulsed ultrasound (LIPUS) is an acoustic wave with a frequency above the hearing threshold of the human ear (20 kHz) and an intensity of 1 to 100 mW/cm^2^ and is commonly used in the field of microenergetic medical therapy.^34^ As this technology continues to develop and mature, more people are also exploring its potential use for more clinical conditions. Recently, new studies have indicated that vascular endothelial dysfunction and the endothelial-type nitric oxide synthase (eNOS)-nitric oxide (NO)-cyclic guanosine monophosphate (cGMP)-PKG pathway may be potential therapeutic targets in the pathophysiology of HFpEF.^33,35,36^ In a series of studies on the improvement of myocardial function by LIPUS, the therapy was able to demonstrate an increase in the expression of angiogenic factors such as eNOS by directly stimulating vascular endothelial cells.^37^ Ultimately, LIPUS has been shown to enhance myocardial perfusion and improve ventricular ejection capacity in animal models of myocardial ischemia and hypertensive heart disease.^38–40^ LIPUS was shown to improve myocardial Ca^2+^-involved processing proteins by enhancing the eNOS-NO-cGMP-PKG signaling pathway, thereby modulating myocardial diastolic function (Figure 3).

In endothelial cells, eNOS is localized in the micropores of the endothelial cell membrane. The endothelial cell membrane is a flow-sensing organelle that converts mechanical stimuli (eg, blood flow-induced shear stress) into chemical signals.^38,41^ The receptor can also sense the cycle strain induced by ultrasonic pulses, leading to the activation of eNOS.^38,42^ Additionally, experiments have indicated that LIPUS can upregulate soluble guanylate cyclase and PKG, the downstream molecules of the eNOS-NO-cGMP-PKG pathway.^37^ By this method, LIPUS can fully activate the eNOS-NO-cGMP-PKG pathway.

Myocardial stiffness associated with myocardial hypertrophy, fibrosis and relaxation ability associated with myocardial Ca^2+^ flux are the influencing factors of cardiac diastolic function. Studies have shown that pharmacological upregulation of the eNOS-NO-cGMP-PKG pathway can effectively improve myocardial hypertrophy and interstitial fibrosis and improve myocardial function.^37,43^ Therefore. When LIPUS fully activates the eNOS-NO-cGMP-PKG pathway, it could probably improve myocardial stiffness and interstitial fiber proliferation to alleviate and treat HFpEF.

At the same time, active relaxation is determined by the energy-dependent removal of Ca^2+^ from the cytosol, and the rate of cytosolic Ca^2+^ clearance during the diastolic phase determines the pattern of muscle relaxation. In contrast, the dynamic levels and processes of Ca^2+^ changes in the cytoplasm are regulated by Ca^2+^-related processing proteins. For example, proteins such as SERCA2a (sarcoplasmic/endoplasmic reticulum Ca^2+^ ATPase 2a) and PLN (phospholamban).^44^ Meanwhile, when the PKG target ser 16 is blocked, cardiomyocyte-specific PKG-KO mice exhibit diminished expression of SERCA2a and PLN and diminished phosphorylation of PLN.^45^ When the eNOS-NO-cGMP-PKG pathway is activated, downstream molecules such as PKG can affect the intracytoplasmic Ca^2+^ clearance of cardiomyocytes by upregulating the expression of Ca^2+^-related processing proteins. This improves the relaxation of cardiomyocytes and restores the diastolic function of the myocardium.

### Invasive Therapy

#### Transcatheter Insertion of an Atrial Shunt

**Figure 4.**
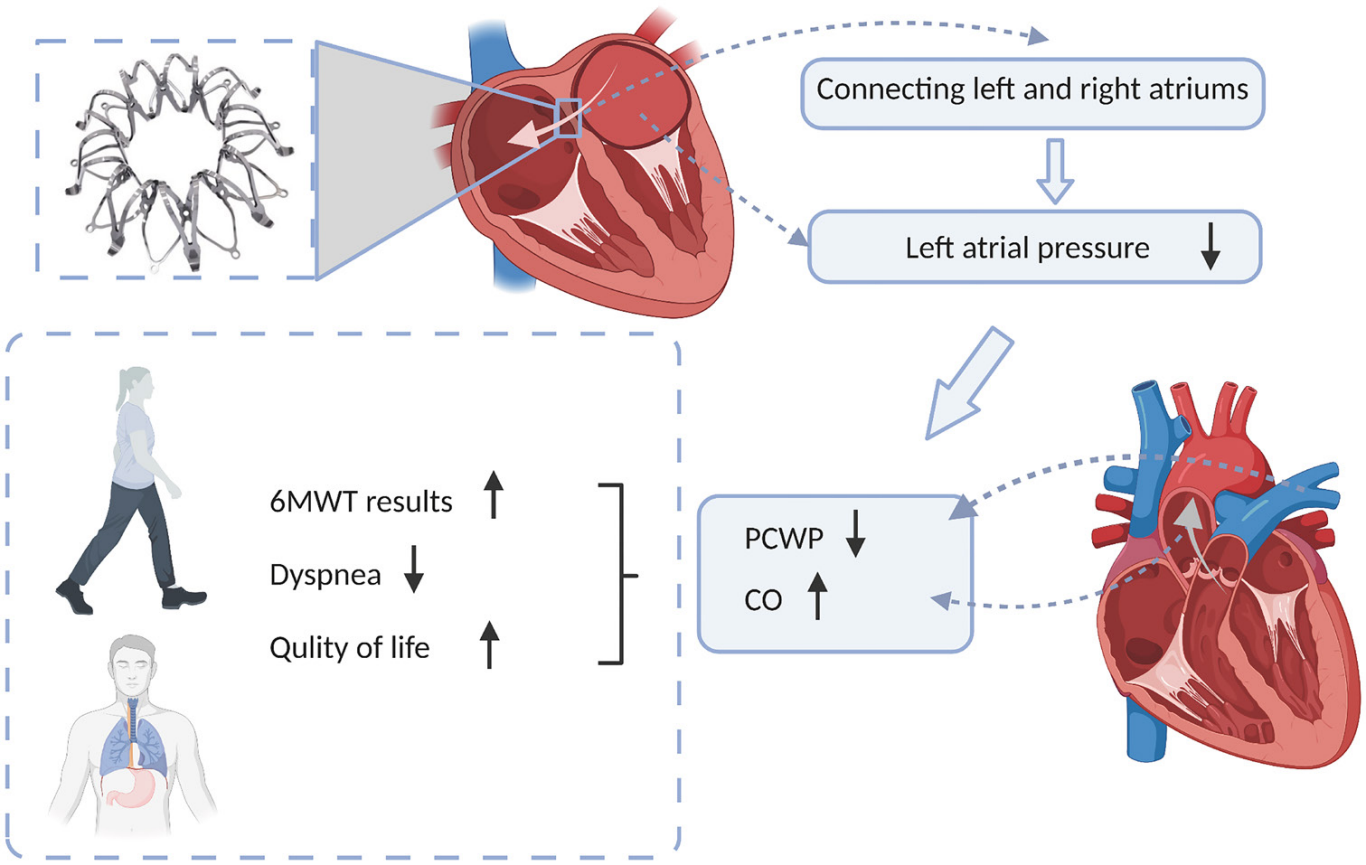
**Septal shunt reduces left atrial pressure and mitigates HFpEF progression.** The septal shunt connects the right and left atria, reducing left atrial pressure and consequently diminishing the elevation of left atrial pressure during HFpEF progression. This leads to a decrease in adverse prognostic outcomes, such as dyspnea, right ventricular dysfunction, and atrial fibrillation. 6MWT indicates 6-minute walk test; CO, cardiac output; HFpEF, heart failure with preserved ejection fraction; and PCWP, pulmonary capillary wedge pressure. Created with Biorender.com.

Interest in atrial shunts for HFpEF was inspired by Lutembacher syndrome, in which the patient’s symptoms of dyspnea due to mitral stenosis were relieved by the decompressive effect of the atrial septal defect.^46^ Previous hemodynamic studies have suggested that medically derived interatrial shunts might reduce left atrial pressure (LAP) and relieve HFpEF-related symptoms.^47^ Currently, there are several devices available for medically derived interatrial bypass, for example, V-Wave (V-Wave Ltd, or Akiva, Israel), an interatrial shunt device (DC Devices Inc, Tewksbury, MA) and the atrial flow regulator (Occlutech, Istanbul, Turkey).^25^ These devices are designed to improve the symptoms of HFpEF by reducing LAP, particularly during activity.^48^ Dynamic and exaggerated increases in LAP are often seen in HFpEF and contribute to dyspnea, atrial fibrillation and impaired right ventricular function.^49^ Therefore, it is natural to envisage that the release of LAP in patients with HFpEF by atrial bypass could alleviate possible clinical symptoms and facilitate prognostic treatment through changes in hemodynamic characteristics (Figure 4). Interatrial shunt devices are safe and practicable and may have short-term clinical utility for patients with HFpEF, according to promising new findings.^46^

To avoid paradoxical embolization and early device closure, the V-wave device has an hourglass-shaped nitinol frame on the left covered by polytetrafluoroethylene polymer and a trilobular porcine pericardial valve on the right.^50^ In a study that was published in 2018, which enrolled 38 patients with HF, including 30 with HFpEF and 8 with HFrEF and exercise pulmonary capillary wedge pressure ≥16 mm Hg, it was discovered that patients using the V-wave device had higher quality of life overall, with their 6-minute walk text increasing by 34 m (*P*=0.02). Among all the hemodynamic parameters obtained, the only one whose change exhibited statistical significance was the shunt ratio Qp/Qs, which increased from unity to 1.17 at 3 months (*P*=0.005). Major adverse cardiovascular and neurological events, such as death, stroke, device embolization and pericardial effusion necessitating intervention, reintervention or surgery, were the primary outcomes assessed at 3 and 12 months of follow-up. The device’s efficacy and safety were confirmed for the first time, providing a clear understanding of its viability in clinical applications.^51^

The interatrial shunt device is a 19-mm-wide metal nickel-titanium alloy frame that is placed through an atrial septal puncture to create an 8-mm hole, which is curved on the right to accommodate septal deformation and flat on the left atrial side to prevent blood clot formation.^52^ Patients with HFpEF (EF >45%) with pulmonary capillary wedge pressure ≥15 mm Hg (rest), ≥25 mm Hg (exercise) and ≥1 hospitalization for HF within the past 12 months or persistent New York Heart Association class III/IV for at least 3 months were included in the REDUCE LAP-HF1 study (Reduced Left Atrial Pressure Elevation in Patients with Heart Failure) to examine the device’s effectiveness and safety.^53^ One year after atrial septal bypass surgery, their short-term outcomes, which were reported in 2016, demonstrated a persistent improvement in the New York Heart Association classification (*P*<0.001), quality of life (Minnesota Heart Failure Life Score, *P*<0.001) and 6-minute walk distance (*P*<0.01). The left ventricular end-diastolic volume index showed a silent but steady drop on echocardiography (*P*=0.001), whereas the right ventricular end-diastolic volume index showed a silent but stable increase (*P*=0.001). Likewise, the mean pulmonary artery wedge pressure showed a decrease of 5 mm Hg in 30 days (*P*=0.005). The improvement in these indices provides evidence for the safety and continued clinical benefit of this device 1 year after the interatrial septal shunt. Further study indicates that the clinical outcome of this treatment modality may be influenced by whether patients have underlying pulmonary vascular disease. In REDUCE LAP-HF II, which included 626 HF (EF ≧40%) patients with pulmonary capillary wedge pressure >25 mm Hg and resting pulmonary vascular resistance <3.5 WU, they defined a cutoff value of pulmonary vascular resistance ≥1.74 WU during exercise as patients with underlying pulmonary vascular disease and found that patients in this group (n=188) had more AF and right heart dysfunction and were more likely to have elevated pulmonary capillary wedge pressure at rest and descending CO during exercise. The worse outcome of atrial shunt therapy in HF patients with latent pulmonary vascular disease suggests the importance of underlying pulmonary vascular disease as a phenotypic marker for individualized treatment.^54^

The atrial flow regulator, which enables interatrial shunting, is a self-expanding double-disc wired mesh with central penetration. Its position is similar to that of an atrial septal defect blocker device.^55,56^ Studies in patients with severe pulmonary hypertension have shown that this device is feasible and effective.^57^ However, the clinical trial application in patients with HFpEF and HFrEF is still unclear, and a preliminary pilot study on this topic was completed in 2021, but the results are not currently published.

#### Personalized Accelerated Pacemaker

In clinical practice, conventional right ventricular pacing plays a crucial role in patients with bradyarrhythmias, such as those with atrioventricular block and sinus node dysfunction. However, as treatment progresses, studies have gradually found that prolonged right ventricular pacing can lead to impaired left ventricular systolic dysfunction in some patients, and the electrical and mechanical dyssynchrony of the myocardium mediated by pacemakers can also increase the risk of patient death. With the introduction of cardiac resynchronization therapy,^58^ it was found that in patients requiring long-term right ventricular pacing, some populations, especially female patients with multiple diseases, are prone to cardiac electrical-mechanical activity asynchrony, and upgrading pacemakers to randomized controlled trials can improve the synchronization of myocardial electrical activity and improve the mechanical function of the patient’s myocardium, potentially reducing the occurrence of adverse prognostic outcomes associated with desynchronization.

Infeld et al conducted a blinded, randomized clinical trial involving 107 patients with HFpEF to investigate whether personalized, accelerated pacemaker settings could reduce the occurrence of negative prognostic outcomes in patients with HFpEF treated with pacemakers. By comparing follow-up data after 1 year with pacemakers using a personalized heart rate derived from a prescribed formula with a factory setting of 60 bpm, improvements were found in Minnesota Living with Heart Failure Questionnaire scores, NT-proBNP (N-terminal pro-B-type natriuretic peptide) levels, atrial fibrillation and adverse clinical events.^59^ The 60 bpm setting dates back to early pacemaker development, was designed to reduce asynchrony associated with right ventricular pacing and is commonly used in pacemaker therapy in patients with HF. However, in patients with HFpEF, although a lower heart rate may improve ventricular filling by prolonging diastolic time, a claim that has not been substantiated, descending heart rate therapy may be detrimental to prognosis. In the large SIGNIFY trial (Study to Assess the Morbidity and Mortality Benefits of the If Inhibitor Ivabradine in Patients with Coronary Heart Disease), selective heart rate lowering with ivabradine (from a mean heart rate of 70–60 bpm) did not improve outcomes but instead led to an increase in adverse clinical events.^60^ This may be related to the lower heart rate causing prolonged ventricular diastole, which in turn leads to increased left ventricular end-diastolic pressure and myocardial hypertrophic remodeling. Therefore, it is expected that with the subsequent confirmation and refinement of further clinical trials, personalized accelerated pacing using heart rates derived based on the characteristics of patients with different HFpEF may become a new idea to improve the prognosis of patients with HF.

#### Cardiac Contractility Modulator

The cardiac contractility modulator (CCM) is an implantable therapeutic device capable of improving myocardial contractility and cardiac function while reversing cardiac remodeling in HF patients.^61^ The only clinically used type is the Optimizer Smart System (Impulse Dynamics) with Food and Drug Administration approval from the United States, which includes a pulse generator that can be recharged and 2 right ventricular active fixation electrodes. By subcutaneous implantation of the extracorporeal programmer and charger device in the chest, the wire is inserted into the right ventricular septum. The basis of this therapy originated from studies in the 1970s that demonstrated that stimulation during the absolute refractory period can stimulate subsequent contractility.^62^ After years of clinical trials to identify its clinical effectiveness and safety, CCM and the theory that it can lower hospital admission rates and enhance exercise tolerance, New York Heart Association functional class and quality of life during follow-up for up to 2 years have been demonstrated in patients with HFrEF.^63–65^ A breakdown of the FIX-HF-5 (Safety and Efficacy Assessment of Optimizer Systems in Subjects with Heart Failure) results per subgroup, the largest clinical trial on CCM to date, found no significant clinical benefit from CCM use in patients with EF <25%, despite the absence of harm, compared with a greater effect in EF >35% to 45%, which may suggest the potential feasibility of its use in patients with HFpEF to improve prognosis. Plus, there is a newly published pilot study that includes 47 patients with preserved ejection fraction, showing it is a promising therapy for the apparent improvement in health status with no influence on safety.^66^ Currently, a cohort study is being conducted to assess the safety and effectiveness of CCM with HFpEF. and is expected to be completed in December 2023 (NCT03240237), offering more support for the clinical application of CCM in HFpEF patients.

**Figure 5.**
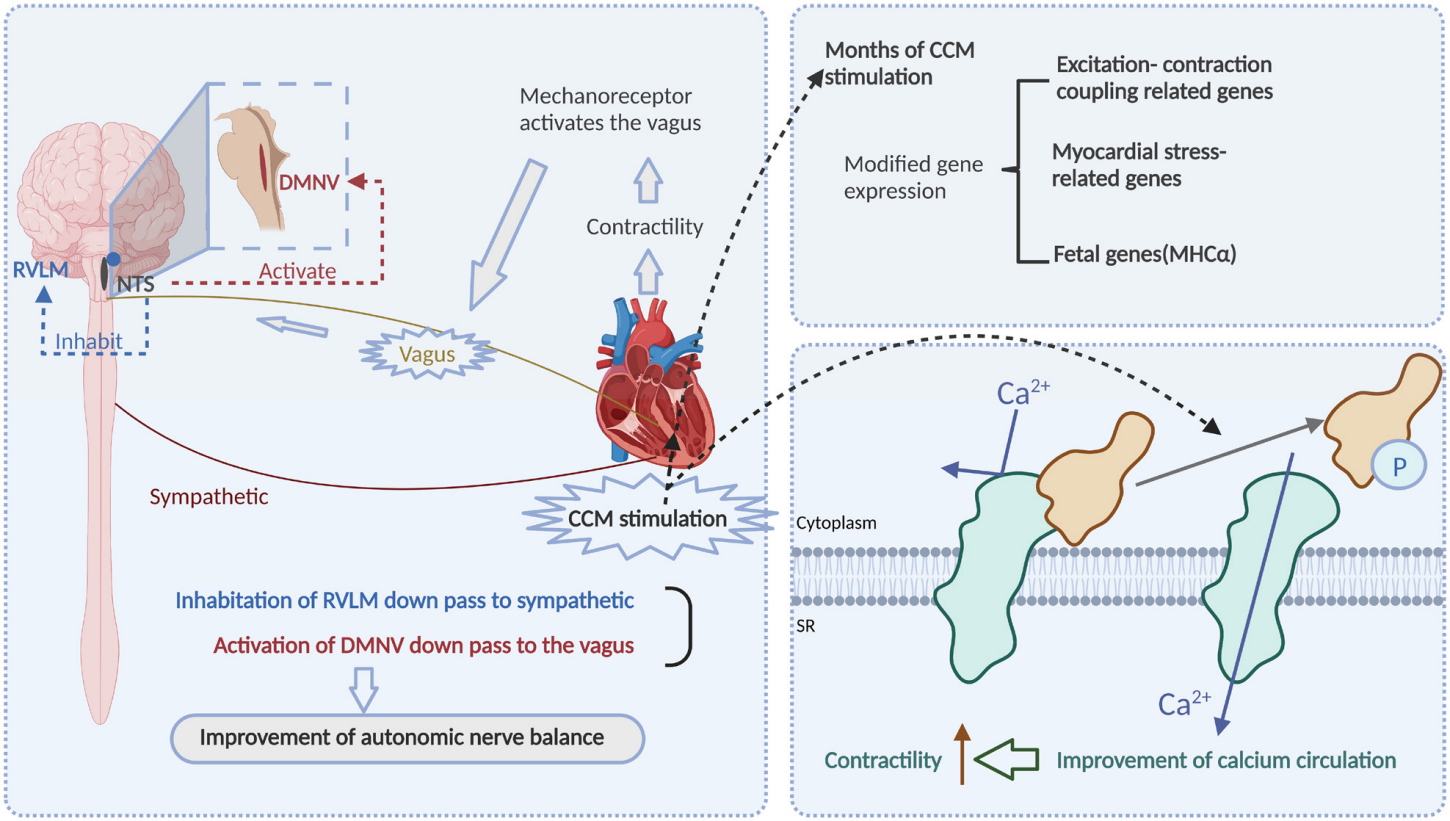
**Atrial contractility regulator mechanism in HFpEF management.** The 3 main aspects of the mechanism are: (1) improved intracellular calcium ion cycling and increased cardiomyocyte contractility through CCM stimulation, (2) enhanced myocardial contractility and autonomic balance, and (3) long-term CCM stimulation that improves gene expression related to excitation-contraction coupling, myocardial stress, and fetal genes (MHCα). CCM indicates cardiac contractility regulator; DMNV, dorsal motor nucleus of the vagus nerve; HFpEF, heart failure with preserved ejection fraction; NTS, nucleus tractus solitarius; P, phosphoric acid; PLB, phosphoprotein receptor; RVLM, right ventral lateral medulla; and SR, sarcoplasmic reticulum. Created with Biorender.com.

The right interventricular septum was subjected to a biphasic (±7.5 V), long duration (20 ms) electrical signal during the absolute refractory period to produce the therapeutic benefit. Although similar to pacemaker therapy, CCM still differs in some aspects, such as the inability of the stimulation provided by CCM to produce myocardial excitation and wavefront propagation and thus exert a pacing effect, and in the specific details of their implantation.^67^ CCM does not affect normal heart rhythm. The strong stimulation provided by CCM is capable of producing positive inotropic effects that improve cardiac health in HF patients without exacerbating myocardial burden and reversing cardiac remodeling with long-term use. The mechanisms by which this positive inotropic effect is produced fall into 3 broad categories: improvement of calcium handling, improvement of autonomic tone, and normalization of pathological HF gene expression.^68^ On the one hand, phosphoproteins are able to inhibit the function of the calcium uptake protein SERCA2a in a nonphosphorylated state. In contrast, CCM is able to phosphorylate phosphoproteins, dissociating phosphoprotein glycans from SERCA2a and enhancing the ability of calcium pumps to reabsorb calcium into the SR, resulting in a positive inotropic effect. On the other hand, CCM stimulation can reduce muscle sympathetic activity through vagal transmission, thus restoring autonomic balance and reducing cardiac output, myocardial work and myocardial oxygen consumption. Finally, CCM treatment is also able to reverse the expression of pathological genes caused by HF, such as ryanodine receptors and SERCA2a, genes responsible for calcium cycling, whose abnormal downregulation can cause abnormal ventricular function and pathological remodeling^69^ (Figure 5).

Currently, CCM is indicated solely for HF patients with an LVEF <45% and in New York Heart Association classes II and III. There is a lack of substantial clinical evidence for its effectiveness in HFpEF patients, necessitating further corroboration through extended trials, which we might gain access to in the near future.

#### Renal Denervation

Renal denervation (RDN) is a technique that blocks the signaling between the central sympathetic nervous system and the kidney, inhibits the activity of the renin-angiotensin-aldosterone system, suppresses the overactivation of the systemic sympathetic nervous system and reduces sympathetic activity by radiofrequency ablation of sympathetic fibers distributed in the vascular wall of the renal artery through a skin catheter. This has been shown to result in durable antihypertensive effects.^70^ There is increasing evidence of a strong correlation between arterial hypertension, vascular stiffness and sympathetic nerve activity.^71–73^ Patients with HFpEF also predominantly exhibit vascular system stiffness, reduced aortic distensibility, increased pulse pressure and increased pulse wave velocity, all of which may be mediated either through elevated sympathetic nerve activity or its long-term mediation.^74^ Therefore, reducing sympathetic nerve activity, improving vascular stiffness and addressing the decrease in ventricular function due to hypertension have become effective ways to treat HFpEF. Hypertension has been shown to improve blood pressure^,75,76^ aortic stiffness^77,78^ and sympathetic nerve activity.^79^ In addition, it can improve the diastolic function of the heart and the left ventricular mass in patients with hypertension^80^ by the following mechanisms.

**Figure 6.**
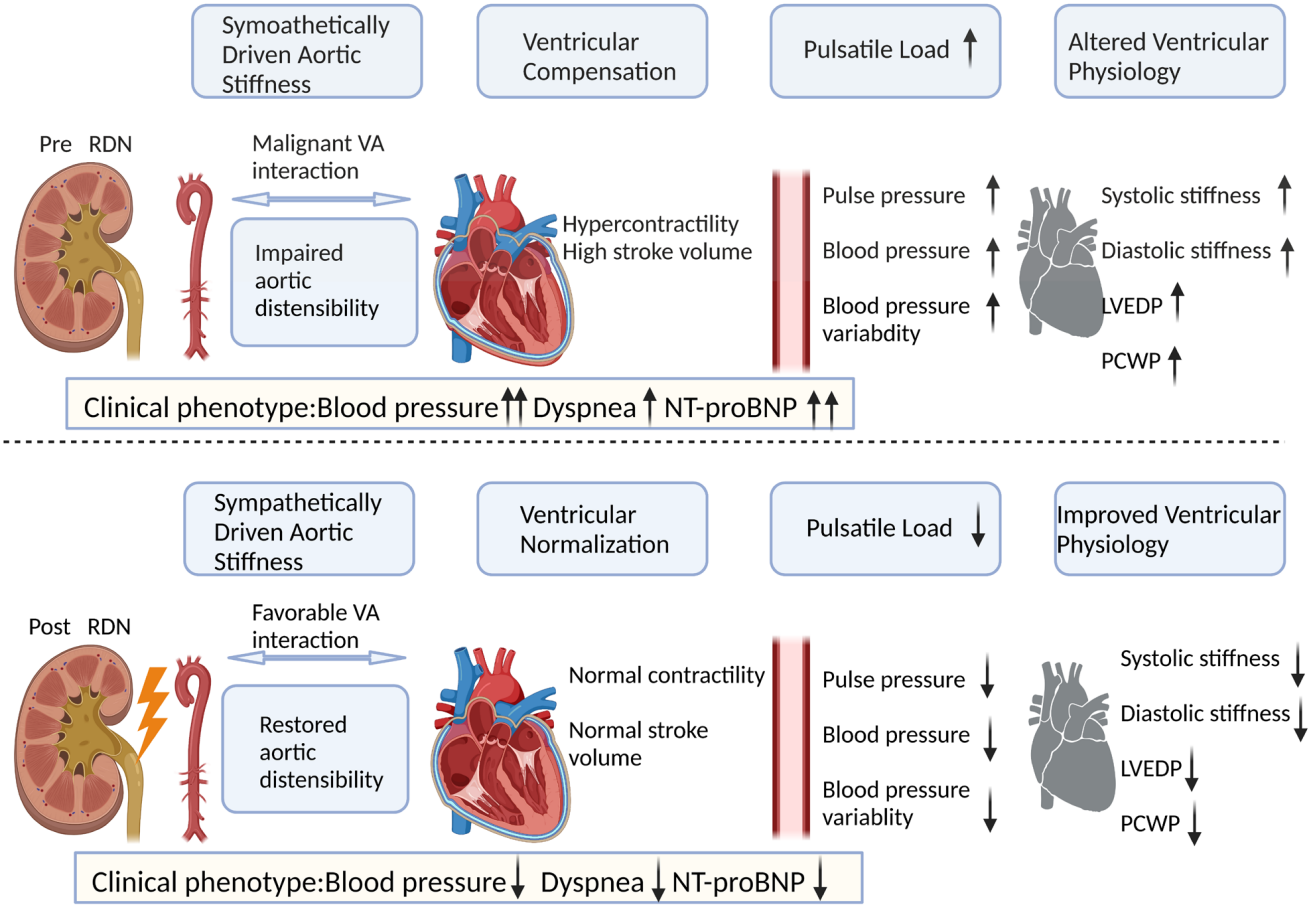
**RDN for ventricular normalization and improved HFpEF outcomes.** RDN reduces aortic stiffness, restores aortic distensibility, and improves ventricular physiology by inducing ventricular normalization. This results in decreased blood pressure, dyspnea, and NT-proBNP levels, ultimately improving ventricular fibrosis and cardiac stiffness. HFpEF indicates heart failure with preserved ejection fraction; LVEDP, left ventricular end-diastolic pressure; NT-proBNP, N-terminal pro-B-type natriuretic peptide; PCWP, pulmonary capillary wedge pressure; RDN, renal denervation; and VA, ventricular arterial. Created with Biorender.com.

The interaction between increased sympathetic activity, which leads to increased aortic stiffness through sympathetic activation, and malignant ventricular arterial compensation, which leads to excessive left ventricular contraction, is an important factor causing HF. When RDN is performed, sympathetic activity is diminished, and the overregulation of the vascular system through the renin-angiotensin-aldosterone system is diminished. This results in a decrease in aortic stiffness and normalization of the ventricles. Subsequently, favorable arteriovenous interaction occurs, contributing to a return of aortic sensitivity and a normalization of contractility and output per beat. By reducing the beat load and improving ventricular physiology through the above process, it is possible to advance pulse pressure and blood pressure and reduce blood pressure volatility. At the same time, systolic stiffness, diastolic stiffness, left ventricular end-diastolic pressure and pulmonary capillary inset pressure were decreased (Figure 6).

The effect of RDN on patients with HFpEF is still in its early stages. Whether it can be extended to all patients with HFpEF needs to be studied more thoroughly. Additionally, which ablation system should be used for RDN and whether one ablation system is better than another needs to be validated and prospectively evaluated.

#### Percutaneous Pulmonary Artery Denervation

Percutaneous pulmonary artery denervation (PADN) is a new technique that improves arterial pressure and promotes the function of the vascular system by damaging the sympathetic nerve of the pulmonary artery and altering it through radiofrequency ablation, ultrasound catheter denervation and open-heart surgery to strip the sympathetic nerve.^81^ Right-sided HF commonly results from the development of pulmonary arterial hypertension (PAH).^82,83^ Although some studies have reported an effect of sildenafil in patients with PAH,^84^ its effectiveness in patients with HF and PH remains somewhat controversial. A recent systematic review and network meta-analysis^85^ suggests that a combination of pharmacological treatments, including ARNI (angiotensin receptor-neprilysin inhibitor), MRAs (mineralocorticoid receptor antagonists) and SGLT2 inhibitors, offers significant therapeutic benefits in reducing cardiovascular death and hospitalizations for HF in patients with HFpEF and HF with mildly reduced ejection fraction. However, for patients with PAH with HFpEF, there are currently no specifically approved pharmacological treatments targeting this particular subgroup. Yet, it has been clinically demonstrated that elevated circulatory catecholamine levels and impaired heart rate variability in patients with HF with PAH are associated with excessive neurohumoral activation,^86–88^ suggesting that precapillary and postcapillary pulmonary hypertension may be treated with local denervation. Additionally, in the study, it was found that treatment with PADN significantly increased the 6-minute walk test.^89–91^ This effect might indicate a significant improvement in cardiac systolic and diastolic function after PADN. Numerous studies on PADN have advanced our understanding of its therapeutic mechanisms to a certain extent. Nonetheless, the precise mechanisms by which PADN exerts its therapeutic effects in the context of HFpEF remain to be fully elucidated.

PADN decreases the nerve conduction function in the pulmonary artery by eliminating local sympathetic nerve damage, attenuates the overactivation of the body’s neurohumoral function due to sympathetic activation, reduces the level of circulating catecholamines and restores heart rate variability. As a result, the kinetics of pulmonary artery hemodynamics were improved, and the sustained recovery of pulmonary artery structures was promoted. Additionally, the consistency of 6-minute walk distance and LV systolic and diastolic function in the study implies a potential role of PADN in LV remodeling. The ultimate therapeutic effect on HFpEF and PAH was achieved.

#### Limitations

In these studies of PADN, the primary end point, the 6-minute walk distance, serves as a functional assessment rather than a direct clinical outcome. Although this measure is informative, it may not fully encapsulate the clinical efficacy of the intervention. Notably, the potential for a learning effect and day-to-day fluctuations in 6-minute walk distance performance could introduce an element of variability that may affect the reliability of our results. Furthermore, the mechanisms by which PADN induces cardiac remodeling have not been completely unraveled. The data we present, while suggestive of PADN’s therapeutic potential, should be considered preliminary and indicative rather than conclusive. The variability in the implementation of the PADN technique, coupled with its varying therapeutic impacts among different patient cohorts, signals a pressing need for further refined research. Collectively, these factors advocate for a conservative interpretation of our findings and underscore the importance of further studies to confirm and expand upon our results.

#### Pericardial Resection

**Figure 7.**
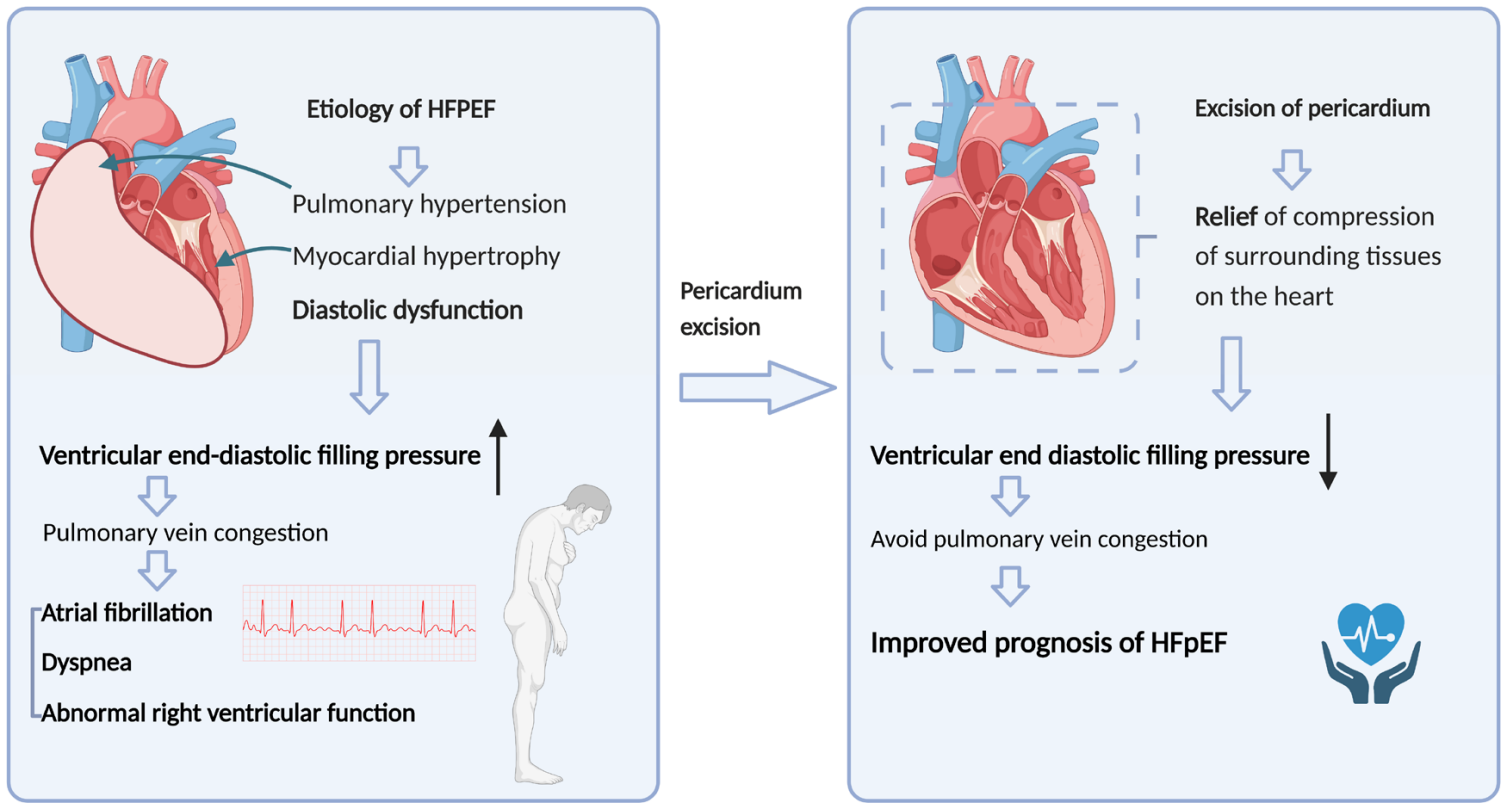
**Pericardial resection mechanism in HFpEF management.** Pericardial resection alleviates compression effects on the heart, reduces ventricular end-diastolic filling pressure, prevents pulmonary vein congestion, and improves HFpEF prognosis by mitigating pathological changes such as pulmonary hypertrophy, myocardial hypertrophy, and diastolic dysfunction that promote HFpEF progression. HFpEF indicates heart failure with preserved ejection fraction. Created with Biorender.com.

Constrictive pericarditis is commonly treated surgically via pericardial excision, and in 2017, it was suggested that minimally invasive pericardial resection could probably become a promising strategy for treating HFpEF.^91^ Currently, there are still relatively few studies related to this approach, with only animal experimental evidence that in both healthy canines and a porcine model with characteristics of HFpEF, pericardial excision using a subxiphoid method promotes improvement of left ventricular diastolic function. In both normal dogs and a porcine model with characteristics of HFpEF, pericardiotomy using a minimally invasive subxiphoid approach with the chest intact improves left ventricular diastolic reserve during saline loading.^91^ Elevated left ventricular filling pressure is a major feature of HFpEF,^92^ while during cardiac diastole, most of the pressure in the left atrium can be attributed to the pressure in the pericardium and the squeezing effect from the right heart.^93,94^ Based on these ideas, pericardial constraints may be removed to improve exercise hemodynamics in HFpEF. Our conjecture about the potential mechanism, informed by relevant studies, is illustrated in the accompanying figure. We propose that in HFpEF, diverse etiologies contribute to a spectrum of pathophysiological changes. These changes include myocardial hypertrophy, pulmonary hypertension and diastolic dysfunction. Pericardial resection, by alleviating ventricular compression, may reduce elevated end-diastolic ventricular filling pressures. Such a reduction has the potential to mitigate the consequences of these pressures, such as pulmonary venous congestion, which can lead to symptoms like dyspnea, as well as complications including atrial fibrillation and compromised right ventricular function. In addition, studies have also shown an improvement in exercise-induced increases in output per beat, cardiac output and maximal exercise capacity in normal animals after pericardiotomy.^95^ These studies suggest that, with a minimally invasive surgical approach and safe prognostic performance, pericardiotomy may be a promising potential nonpharmacological treatment for HFpEF. Nevertheless, research on this treatment is currently limited to a few animal studies, as animal models of HFpEF simulate only some features of HFpEF, including elevated left ventricular end-diastolic pressure, hypertension, oxidative stress and coronary microvascular dysfunction,^96–99^ which may not be representative of actual clinical patient outcomes, and experiments have used saline loading to simulate left ventricular filling. The results of the study are still unclear as to whether pericardial resection in clinical HFpEF patients can actually reduce LV filling pressures and thus lead to improvements in indicators such as exercise capacity and dyspnea status, as well as the long-term safety of this surgical approach, and more in-depth studies are urgently needed to determine this (Figure 7).

### Comorbidity Treatment

#### Comorbidity and HFpEF

**Figure 8.**
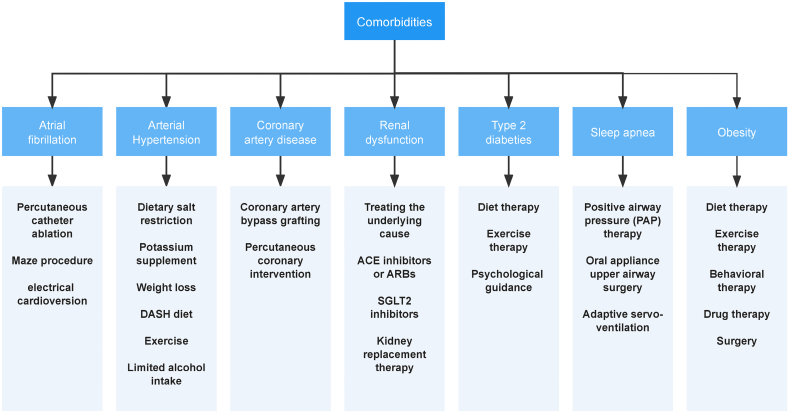
**HFpEF comorbidities and associated nonpharmacological therapies.** This illustration summarizes the key comorbidities of HFpEF and their corresponding nonpharmacological treatment options. HFpEF indicates heart failure with preserved ejection fraction.

In addition to the complex pathophysiological mechanisms of HFpEF, the multiple comorbidities that often accompany it and the interactions with HFpEF are possible factors in the lack of effective treatments currently available. A variety of conditions are associated with HFpEF (shown in Figure 8).^49^ Notably, all of the above comorbidities are highly prevalent in the elderly,^100^ and the increasing proportion of elderly people in the global population will lead to a further increase in the prevalence and incidence of HFpEF in the future. Despite the advancement in HF treatment with the introduction of SGLT2 inhibitors, mineralocorticoid receptor antagonists and other pharmacological treatments supported by current evidence,^3^ managing the wide array of comorbidities in patients with HFpEF remains a pivotal aspect of improving patient outcomes. This approach underscores the necessity of a comprehensive treatment strategy that addresses the multifaceted nature of HFpEF.^101^ Not only is the prevalence of noncardiac comorbidities higher compared with HFrEF, but the impact on HF prognosis by treating comorbidities is also more pronounced in patients with HFpEF,^102^ which illustrates the clinical significance of nonpharmacological treatment for comorbidities on the prognosis of HFpEF. Here, we mainly exploit the potential in HFpEF patients with obstructive sleep apnea (OSA) and obesity, the 2 sorts of cases.

#### Treatment of OSA

OSA is a condition with a relatively high prevalence in HF patients. It appears that there are many similarities between the pathological effects of OSA and other known etiologies of HFpEF and its postulated pathophysiology. OSA can reduce intrathoracic pressure and promote venous return, which in turn leads to a left-ward septal shift, causing a decrease in stroke volume. Along with hypoxia induced by OSA, this leads to the stimulation of the sympathetic nervous system, renin-angiotensin-aldosterone system and, more importantly, a systemic inflammatory state, therefore probably causing myocardial fibrosis and structural changes in collagen and myocardial titin that cause myocardial stiffening.^103^

However, the clinical effects on diastolic dysfunction of using continuous positive airway pressure in HFpEF patients are mixed. In a trial involving 36 patients with HF, LVEF >50% and an apnea-hypopnea index >15 randomized to 6 months of adaptive servo-ventilation and then followed for a further year, symptom class, left atrial volume, B-type natriuretic peptide concentration, ventricular filling and event-free survival all improved in those so treated.^104^ On the contrary, a randomized trial that followed a subgroup of 2717 eligible adults aged 45 to 75 years with obstructive sleep apnea and cardiovascular or cerebral conditions concluded that, compared with usual care alone, continuous positive airway pressure ventilation plus usual care treatment did not prevent cardiovascular events in those patients.^105^ To overcome the limitation of the scarceness of the relevant study, a large-scale randomized study of continuous positive airway pressure in the HFpEF population with OSA is expected.

#### Treatment of Obesity

With the fact that more HFpEF patients have various levels of obesity, even if they still have visceral adiposity, it is natural to pay attention to this condition as well as its role in the formation and progression of HFpEF and clinical feedback after treatment. Obesity worsens HFpEF outcomes by exerting adverse effects on the cardiovascular system, including pulmonary and right ventricular function, multiple biomarkers for inflammatory signaling and metabolic disease.^106^ Besides, there could be right ventricular dysfunction in severe obesity patients with HFpEF.^107^ For patients with obesity and HFpEF, modest (ie, 6.6%) weight loss induced through caloric restriction (prepared meals to achieve a reduction in energy intake by 400 kcal/d) improved functional status (change in peak oxygen consumption of 1.3 [95% CI, 0.8–1.8] mL/kg·min compared with attention control; n=100).^3^ Another landmark trial showed that, besides through prepared meals, aerobic exercise could also reduce LV mass and inflammatory markers, improve exercise capacity, and enhance quality of life in patients with obese HFpEF.^108^ Clinical data on weight loss through surgery and the use of medications are yet to be supplemented.

## CONCLUSIONS

Despite ongoing challenges in identifying evidence-based treatments with clear prognostic improvements for HFpEF, nonpharmacological approaches are increasingly gaining scholarly attention and show promise in addressing this unmet need. This review has summarized the latest advancements in nonpharmacological treatments for HFpEF, highlighting the potential benefits of various techniques. VNS, PADN and RDN have been shown to reduce ventricular pressure and improve HFpEF outcomes by modulating nervous system overregulation. Additionally, interventions such as atrial shunt devices and pericardial resection can enhance HFpEF prognosis by altering cardiac structure and directly decreasing heart filling pressure. On the contrary, CCMs improve myocardial contractility through direct external stimulation, leading to gradual functional enhancements. Treating comorbidities, including hypertension and diabetes, in certain patients with HFpEF has also demonstrated beneficial effects on disease outcomes. These innovative techniques, combined with well-established exercise therapies, signify significant progress in HFpEF management and lay the groundwork for future research and clinical applications. As our understanding of these treatment mechanisms deepens, the utilization of these techniques and approaches is expected to enable comprehensive HFpEF management.

## ARTICLE INFORMATION

### Sources of Funding

This study was supported by the Clinical Medical Technology Innovation Guidance Project of Hunan Science and Technology Agency (2021SK53519) and the Natural Science Foundation of Hunan Province, China (2023JJ30791).

### Disclosures

None.
